# Transposable elements reveal a stem cell-specific class of long noncoding RNAs

**DOI:** 10.1186/gb-2012-13-11-r107

**Published:** 2012-11-26

**Authors:** David Kelley, John Rinn

**Affiliations:** 1 Department of Stem Cell and Regenerative Biology, Harvard University, Cambridge, MA 02138, USA; 2 Broad Institute of Massachusetts Institute of Technology and Harvard, Cambridge, MA 02142, USA; 3 Department of Pathology, Beth Israel Deaconess Medical Center, Boston, MA 02215, USA

## Abstract

**Background:**

Numerous studies over the past decade have elucidated a large set of long intergenic noncoding RNAs (lincRNAs) in the human genome. Research since has shown that lincRNAs constitute an important layer of genome regulation across a wide spectrum of species. However, the factors governing their evolution and origins remain relatively unexplored. One possible factor driving lincRNA evolution and biological function is transposable element (TE) insertions. Here, we comprehensively characterize the TE content of lincRNAs relative to genomic averages and protein coding transcripts.

**Results:**

Our analysis of the TE composition of 9,241 human lincRNAs revealed that, in sharp contrast to protein coding genes, 83% of lincRNAs contain a TE, and TEs comprise 42% of lincRNA sequence. lincRNA TE composition varies significantly from genomic averages - L1 and Alu elements are depleted and broad classes of endogenous retroviruses are enriched. TEs occur in biased positions and orientations within lincRNAs, particularly at their transcription start sites, suggesting a role in lincRNA transcriptional regulation. Accordingly, we observed a dramatic example of HERVH transcriptional regulatory signals correlating strongly with stem cell-specific expression of lincRNAs. Conversely, lincRNAs devoid of TEs are expressed at greater levels than lincRNAs with TEs in all tissues and cell lines, particularly in the testis.

**Conclusions:**

TEs pervade lincRNAs, dividing them into classes, and may have shaped lincRNA evolution and function by conferring tissue-specific expression from extant transcriptional regulatory signals.

## Background

Recent comprehensive transcriptome sequencing studies uncovered a large class of previously unannotated long noncoding RNA (lncRNA) genes in various species with similar splicing and polyadenylation properties to mRNAs [1-8]. Genome-wide analyses found that human lncRNAs are more tissue-specific than protein coding genes and are preferentially proximal to developmental regulators [2,9]. Accumulating evidence suggests lncRNAs are key regulators in cell differentiation and disease pathways [10-18].

Initial progress has been made to understand the evolution and origins of lncRNAs [6]. The nucleotide-level conservation of lncRNAs is well-studied in vertebrates using simple substitution and indel-based models, which suggest that lncRNAs are more conserved than neutrally evolving regions of the genome, but less conserved than protein coding genes [1,2,19,20]. Though extensive lncRNA catalogs have been discovered in diverse organisms, recently including zebrafish [3,4], *Drosophila *[5], and nematode [21], distant homologues to human lncRNAs are less frequent and more diverged than protein coding gene homologues [2-4,21,22]. Collectively, these studies suggest that while many species have numerous lncRNAs, they rapidly evolved in a species-specific manner or exhibit other mechanisms of evolutionary constraints.

One important method by which the genome, including lncRNA sequence, evolves is transposable element (TE) insertions. TEs are nucleic acid sequences capable of inserting into genomic DNA that are typically considered 'selfish' genomic parasites and have conquered 45 to 65% of the human genome [23,24]. Despite the selfish origins of TEs, their activity occasionally has subtle evolutionary benefits [25,26], which has allowed TEs to significantly shape the evolution of the human genome [27].

In a few known cases, TE proteins required for transposition have seeded novel genes in the host genome [28-31]. More often, TEs influence transcriptional regulatory networks. For example, TE promoters, particularly the long terminal repeats (LTRs) of endogenous retroviruses (ERVs), initiate transcription at some protein coding genes, typically as alternative promoters [32-34]. Further, TEs have shaped gene regulation by distributing transcription factor binding sites [35-39], spawning enhancers [40,41], and possibly by composing highly conserved noncoding regions [42,43]. In addition to proteins, ncRNA genes, particularly microRNAs [44], can be derived from TEs [45]. Post-transcriptionally, Alu elements (and potentially other TEs) harbor splicing signals, and insertions in protein coding genes have created new splice sites and exons [46-49]. Taken together, these studies demonstrate extensive shaping of gene regulatory networks by TE insertions.

Whether TEs have similarly influenced lncRNA sequence and regulation is largely unexplored, but numerous recent studies point to interesting TE-associated lncRNA functions. For example, Alu elements in lncRNAs play a significant role in STAU1-mediated mRNA decay by duplexing with complementary Alu elements in the 3' UTRs of mRNAs [50]. A mutated L1 element in a lncRNA is associated with infantile encephalopathy [51]. We previously identified ten lncRNAs that were significantly upregulated in induced pluripotent stem cells (iPSCs) relative to human embryonic stem cells (ESCs) [52]. Seven of these ten lncRNAs, including one that was required for reprogramming (*linc-ROR*), have HERVH elements near the 5' transcript end, suggesting HERVH elements may shape lncRNA regulation in the pluripotent state.

Here we comprehensively characterize the TE composition of long intergenic noncoding RNAs (lincRNAs) and their functional relationships in the human genome. We find that lincRNAs contain TEs at a far greater rate than protein coding genes and are highly enriched for ERVs and depleted of LINEs and SINEs. TEs have position and orientation preferences in lincRNAs, including a frequent association of LTRs with lincRNA transcription start sites (TSSs) that suggests a role in the genes' origins. In a number of intriguing cases, TE content correlated with lincRNA expression properties. Strikingly, lincRNAs containing HERVH elements exhibit a stem cell-specific expression pattern. These results demonstrate that lincRNAs have nonrandom composition of TEs that strongly correlates with their functional and regulatory properties, suggesting a mechanism for malleable evolution of lincRNAs.

## Results

### Human reference catalogs of lincRNAs and TEs

To investigate the relationship between lincRNAs and TEs, we first established a reference catalog of TEs in the human genome from RepeatMasker annotations of Hg19. Removing non-TE repeats left 4.5 million TEs, covering 49.9% of the genome. Next, we assembled a catalog of human lincRNAs from RNA sequencing (RNA-Seq) of 28 different tissues and cell lines using methods from our previous human lincRNA annotation effort [2] with careful processing of multi-mapping reads (Materials and methods). We filtered transcripts assembled from these data to remove those associated with protein coding genes, leaving 9,241 lincRNAs (Materials and methods). A thorough analysis of the genes determined that our updated lincRNA catalog is consistent with one recently published (Figure S1 in Additional file 1) [2].

### lincRNAs comprise a nonrandom distribution of TEs

**Figure 1:**
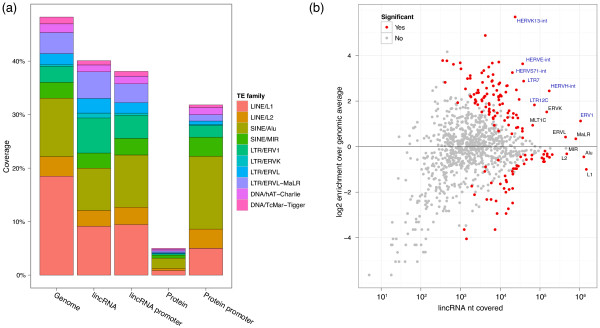
**Transposable element composition of human lincRNAs**. We intersected TE annotations with a catalog of 9,241 human lincRNAs. **(a) **TEs compose less lincRNA sequence than genomic background but much more than protein coding genes. Promoters for the two gene classes are more similar than the transcripts. **(b) **The lincRNA frequencies of many specific TE families differ significantly (based on a shuffling statistical test) from their genomic averages. Larger families are to the right. Enrichments are above zero on the y-axis, and depletions are below zero. ERV1 families (labeled in blue) are particularly enriched.

Intersection of the lincRNA and TE catalogs revealed that the vast majority (7,710, 83.4%) of lincRNAs overlap at least one TE. In fact, nearly half (41.9%) of lincRNA transcript sequence is TE-derived (Figure 1a; Figure S2 in Additional file 1; Additional file 2). In sharp contrast, TEs overlap only 6.2% of protein coding sequences and cover 0.32% of their nucleotides (Additional file 3). Including UTRs, these numbers increase to 39.1% of protein transcripts overlapping TEs and 5.5% of sequence covered. The median proportion of TE-derived sequence among lincRNAs is 33%, and there are 2.8 unique TE families per lincRNA on average (Figure S3 in Additional file 1).

lincRNAs exhibit many biases in their TE composition relative to genomic averages (Figure 1b; Additional file 2). The LINE L1 and SINE Alu families are the most prevalent in the human genome, together accounting for 29% of genomic sequence. Though also the most prevalent TE families in lincRNAs, both are significantly depleted, L1 by 2.0-fold (*P *4e-134) and Alu by 1.4-fold (*P *1e-29). Other common LINE and SINE families, L2 and MIR, as well as DNA transposons hAT-Charlie and TcMar-Tigger, are also significantly depleted.

Conversely, retroviral elements ERV1, ERVL-MaLR, ERVL, and ERVK are enriched in lincRNAs (Figure 1b). ERVs are remnants of exogenous retrovirus insertions into the germline and contain deteriorating retroviral protein open reading frames, flanked by transcription-promoting LTRs [53]. The ERV1 family occurs 2.2-fold more in lincRNAs (*P *2e-140) and makes up the most lincRNA sequence of these families.

**Figure 2:**
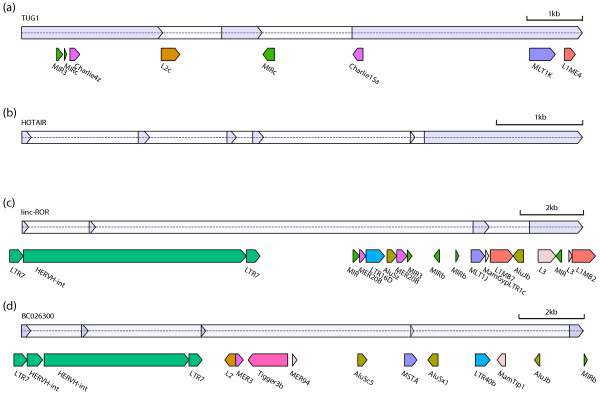
**Example lincRNAs with TE annotations**. lincRNA exons are drawn above in blue, with introns colored lighter. TEs are colored by family, matching the legend in Figure 1a. **(a) ***TUG1 *serves as a typical example of a lincRNA containing multiple TE families. **(b) **Alternatively, *HOTAIR *and 1,531 (17%) of the lincRNAs in our catalog are devoid of TEs. **(c) ***Linc-ROR *is almost entirely composed of TEs, including its TSS in the LTR of a HERVH element. **(d) ***BC026300 *also initiates transcription in a HERVH. The images were created using the software AnnotationSketch [93].

Figure 2 displays the TE composition of several example lincRNAs. The lincRNA *TUG1 *interacts with methylated Polycomb 2 protein to modulate its recognition of histone modifications [54,55] and serves as an example of typical multi-family TE composition (Figure 2a). Alternatively, the lincRNA *HOTAIR*, located in the HOXC cluster, a genomic region known to be nearly devoid of TEs [23], is one of 1,531 lincRNAs without any TE-derived sequence (Figure 2b). *Linc-ROR*, which modulates reprogramming of fibroblasts to a pluripotent state, is almost entirely composed of TE-derived sequence from seven different TE families and has an ERV1 LTR at its TSS (Figure 2c). The HERVH element at the TSS of *linc-ROR *is a common phenomenon in our lincRNA catalog, elaborated on below and depicted again for UCSC-annotated *BC026300 *(Figure 2d).

### Properties of lincRNAs containing TEs

We next investigated the basic properties of lincRNAs containing TEs relative to those that do not. We refer to the set of 7,710 lincRNAs that overlap a TE as TE-lincRNAs and the 1,531 that are devoid as dTE-lincRNAs. Similarly, when discussing a particular TE family, such as L1, we use L1-lincRNAs to refer to the set of lincRNAs containing an L1 element. All analyses were also performed for mRNAs. Here, we focus mainly on those properties that are unique to lincRNAs relative to mRNAs.

#### Transcript structure

lincRNAs with TEs are larger than those without (*P *1e-168; Figure S5a in Additional file 1), an expected difference because larger lincRNAs present more sequence for TE insertions. TE-lincRNAs have geometric mean length of 1,179 versus 599 for dTE-lincRNAs. TE-lincRNAs also have greater splicing complexity than dTE-lincRNAs (Figure S5b, c in Additional file 1), with 2.88 versus 2.59 exons/transcript (*P *2e-27) and 2.35 versus 2.09 isoforms/gene (*P *1e-49). The correlation between transcript length and these splicing complexity statistics is weak and insufficient to explain the difference.

Though depleted relative to genomic averages, TEs are far more prevalent at lincRNA splice junctions (donor 30.8%, acceptor 33.8%) than mRNA splice junctions (donor 0.79%, acceptor 0.76%). Previously observed for proteins, MIRs are enriched at splice sites relative to the transcript sequence of both lincRNAs (donor 1.4-fold, acceptor 1.3-fold) and proteins (donor 2.3-fold, acceptor 1.4-fold) [56]. Taken together, these results suggest TEs have influenced lincRNA transcript structure.

#### Gene expression

Next, we examined the expression patterns of lincRNAs with respect to their TE composition. To this end, we analyzed lincRNA abundance estimates across an RNA-Seq database of the 28 tissues and cell lines used for assembly along with additional iPSC RNA-Seq (a total of > 4 billion reads; Additional file 4). We estimated gene abundance (measured as fragments per kilobase per million fragments (FPKM)) using Cufflinks and took additional measures to minimize potential artifacts of multi-mapping reads on the abundance estimates (Materials and methods) [57]. Using this compendium, we compared the expression patterns of lincRNAs containing various classes of TEs.

We observed several intriguing expression biases based on lincRNA TE composition. In every tissue and cell line, TE-lincRNAs were less expressed than dTE-lincRNAs, with nearly all of the differences statistically significant. This observation is not confounded by the difference in length between the two gene classes (Figure S7 in Additional file 1). The expression divergence in testis was the most striking and significant (*P *3e-10; Figure S6a in Additional file 1). Despite the relative difference, FPKM values of both TE- and dTE-lincRNAs rank highest in testis over all other tissues and cell lines, consistent with previous observations of widespread transcription in testis [58]. In sharp contrast to TEs overall, the presence of an Alu element correlates with greater expression in all tissues and cell lines except testis (*P *3e-8; Figure S6b in Additional file 1).

Previously, lincRNAs were observed to be far more tissue-specific than protein coding genes [2]. As in Cabili *et al. *[2], we define tissue specificity as a function of the Jensen-Shannon divergence between expression profiles. Overall, the tissue specificity of TE-lincRNAs and dTE-lincRNAs is similar, despite their abundance differences. However, Alu-lincRNAs are less tissue-specific (*P *6e-57), refining our observation above that Alu-lincRNAs are more expressed in all tissues but testis. Collectively, these results suggest an intriguing relationship between lincRNA TE composition and expression patterns.

#### Conservation

Though less conserved than protein coding genes, lincRNAs are more conserved than neutrally evolving sequence by traditional substitution-based statistics [2,59-61]. Furthermore, prior analysis of a mouse lincRNA catalog concluded that TEs within lincRNAs are no more conserved than those genome-wide [59]. Working towards a better understanding of the functional significance of TEs in human lincRNAs, we analyzed lincRNA mutation patterns through the more refined lens of TE annotations using PhastCons and PhyloP conservation scores assigned based on the placental mammal phylogeny [62,63].

Consistent with observations in mouse [59], conservation of TEs in lincRNAs is low and nearly indistinguishable from that of TEs genome-wide (Figure S8a in Additional file 1). We next explored the relationship between lincRNA TE composition and conservation by comparing conservation scores between TE- and dTE-lincRNAs. Strikingly, we found that dTE-lincRNAs are far more conserved, with mean PhastCons conservation probability 16.0% versus 8.0% for TE-lincRNAs (*P *~ 0; Figure S8b in Additional file 1). Thus, these 1,531 lincRNAs experience strong negative selection against both nucleotide substitutions and TE insertions.

To explore the degree to which the lower conservation of TE-lincRNAs is driven by the TE sequence itself, we compared the conservation scores of TE and non-TE sequence in these genes. The non-TE sequence has slightly greater conservation, with mean PhastCons probability 8.5% versus 7.6% for TE sequence, but still less than that of dTE-lincRNAs. Statistical significance of this comparison is challenging due to the widely different distribution shapes, which is viewed most clearly in the substantially decreased variance in PhyloP scores assigned to TE sequence - most are near zero (Figure S8c in Additional file 1). This pattern suggests a scarcity of alignments to other mammalian genomes, unsurprising for these repetitive and often lineage-specific elements. Overall, these results highlight the conservation of dTE-lincRNAs, while indicating that TE-lincRNAs mutate more freely.

#### TE position and orientation biases in lincRNAs

Based on the intriguing enrichment of LTRs at lincRNA TSSs, we hypothesized that TEs have influenced lincRNA transcriptional regulation, similarly to protein coding genes [32-34]. In search of evidence, we analyzed TE position and orientation within lincRNA gene loci. For each TE family, we plotted its coverage around the 5' and 3' ends of all lincRNAs. To examine coverage in the lincRNA interior, we divided each lincRNA into 100 uniformly spaced bins and plotted TE coverage of the bins. In addition, we looked for biases from a null model of 50/50 sense/antisense orientation of the TEs with respect to the lincRNAs they compose (Additional file 5).

Our analysis revealed several TE position biases in lincRNA loci. For example, Alu elements exhibit a distinctive peak approximately 250 nucleotides downstream of the 3' ends of lincRNAs (Figure S9 in Additional file 1), where they appear more often in the sense orientation (61%, *P *2.7e-7). AluY drives the 3' peak and is sense oriented in 71% (*P *4.7e-4) of the 108 lincRNA 3' ends that it marks. This observation is consistent with the known role of Alu elements in contributing polyadenylation signals to the 3' ends of many protein coding genes [64,65]. In our data, Alu elements, and AluY in particular, exhibit similarly biased orientation at the 3' ends of protein coding genes (81%, *P *6e-47), albeit without a coverage peak (Figure S9 in Additional file 1).

At lincRNA TSSs, both LINE families L1 and L2 tend to be oriented antisense (*P *2.6e-6 and 1.2e-7, respectively). This suggests a minor role for the L1 antisense promoter in initiating lincRNA transcription, which has been documented for protein coding genes [66]. The various L1PA families are most responsible for this effect - 91% of the 53 full-length elements at a TSS are antisense to the lincRNA (*P *2.6e-6).

**Figure 3:**
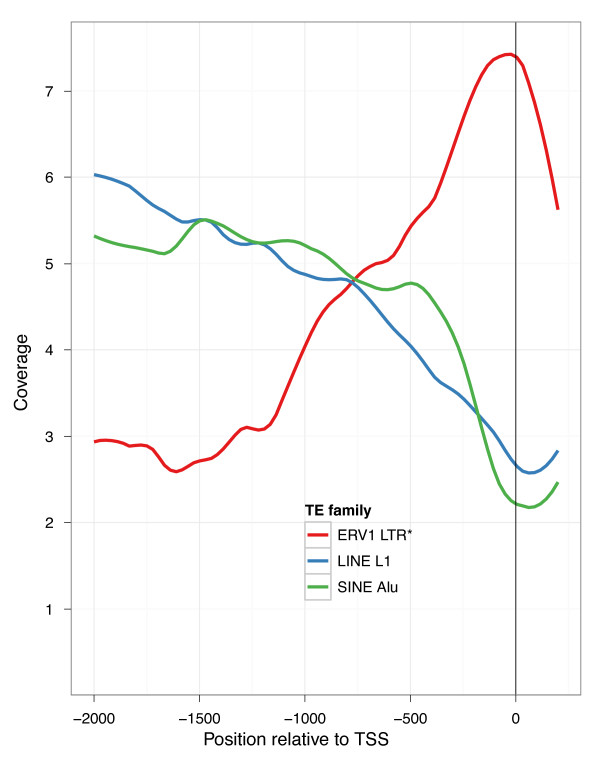
**ERV1 LTRs associate with lincRNA TSSs**. We plotted the coverage of various TE families approaching lincRNA TSSs. The prevalent L1 and Alu families are depleted in lincRNAs. Accordingly, their coverage drops throughout lincRNA promoters leading up to the TSS. Alternatively, ERV1 elements are enriched in lincRNAs, and coverage of the transcription-promoting ERV1 LTRs peaks at the TSS. This pattern was not observed for mRNAs (Figure S11 in Additional file 1).

Further substantiating our hypothesis that ERVs have influenced lincRNA transcriptional regulation, we found that all ERV families significantly prefer the sense orientation with respect to lincRNAs. Driving this bias is an association between LTRs, which are known to harbor promoter signals, and TSSs. ERV1 LTRs occur 72% in the sense orientation at TSSs (*P *4e-12) and have a large coverage peak directly at the TSS (Figure 3). Three prevalent ERV LTR families epitomize this association throughout the genome - ERV1 elements LTR7 and LTR12 and ERVL-MaLR element THE1. Each family is enriched at lincRNA TSSs, peaks in coverage at the TSS, and prefers the sense orientation (Figure S10 in Additional file 1). In contrast, these ERV LTRs are severely depleted at protein coding gene TSSs (Figure S11 and S12 in Additional file 1). However, their few occurrences have the same orientation bias (22 sense, 2 antisense), suggesting that they may also serve as regulatory factors in the promoter regions of these few protein coding genes.

Intrigued by the possibility that these ERV insertions mark the originating event for these many lincRNAs, we conducted additional analysis of their TSSs. Because we chose the most expressed isoform from each gene loci, we have focused the analysis towards the primary TSS rather than a weak alternative TSS, as LTRs have been found to mark in protein coding genes [32-34]. Furthermore, in a stringent set of 298 lincRNAs with an ERV LTR in the sense orientation directly at their TSS, 65% have only that single start site for all isoforms. For these lincRNAs, there is considerable evidence that the ERV insertion originated the gene; at least, it significantly shaped transcription at the loci.

In summary, lincRNAs exhibit biased position and orientation at transcript endpoints. In particular, ERV LTR patterns at lincRNA TSSs suggest that TEs may have originated and imparted regulatory signals to lincRNAs.

#### HERVH elements associate with stem cell-specific expression of lincRNAs

**Figure 4:**
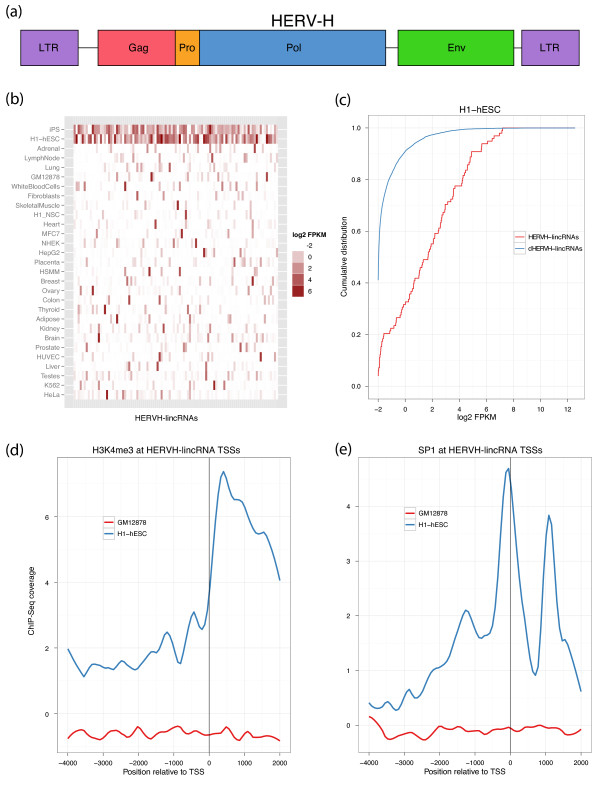
**HERVH elements associate with stem cell-specific lincRNA expression**. **(a) **HERVH is a primate-specific 9 kb endogenous retrovirus containing the group specific antigen (Gag), protease (Pro), polymerase (Pol), and envelope (Env) proteins, surrounded on both sides by transcription-promoting LTRs. **(b) **127 lincRNAs (columns) contain HERVH elements and expression of these lincRNAs (measured as log2(FPKM + 0.25)) across cell types (rows) is highly specific to the pluripotent H1-hESCs and iPSCs. **(c) **HERVH-lincRNAs are expressed at much greater levels than lincRNAs devoid of HERVH (dHERVH-lincRNAs) in ESCs, displayed here as the cumulative distribution of FPKM + 0.25. **(d) **ChIP-Seq read coverage indicates that HERVH-lincRNAs are marked by the activating histone modification H3K4me3 in H1-hESCs but not GM12878 where expression is low. **(e) **The transcription factor SP1 was previously found to be required for HERVH transcription. Accordingly, ChIP-Seq read coverage shows SP1 occupies the TSSs of HERVH-lincRNAs in H1-hESC but not GM12878.

The most significantly enriched individual TE family in our human lincRNA catalog is the human endogenous retrovirus H (Figure 1b). HERVH is annotated by RepeatMasker as an interior component HERVH-int, flanked on both sides by LTR7 (Figure 4a). Further piquing our interest, we previously observed HERVH in the promoter regions of seven out of ten lincRNAs highly expressed in iPSCs relative to ESCs, including *linc-ROR*, which modulates reprogramming [52]. Strikingly, we found that the 127 HERVH-lincRNAs in our catalog are expressed at much higher levels in pluripotent cells, H1-hESCs and iPSCs, than any other tissue or cell line (Figure 4b). Rank sum statistical tests comparing expression of lincRNAs with and without specific TE elements highlighted HERVH-lincRNAs in H1-hESC and iPSC with 8.3- and 4.3-fold greater FPKM geometric means over lincRNAs devoid of HERVH (*P *5e-37, 3e-39; Figure 4c). This property is specific to lincRNAs - it does not apply to 5 mRNAs for which the primary isoform overlaps HERVH, nor 30 mRNAs with a HERVH up to 2 kb upstream (Figure S13 in Additional file 1).

As alluded to and exemplified in Figure 2c, d, HERVH shows a strong preference for the sense orientation at lincRNA TSSs. LTR7 coverage rises sharply, starting approximately 500 nucleotides upstream and peaking directly at the TSS (Figure S10a in Additional file 1). In the lincRNA interior, LTR7 coverage subsequently drops, verifying that the pattern is truly a peak rather than the boundary of a coverage plateau (Figure S10a in Additional file 1). Given the propensity of LTRs to act as promoters, it is plausible that HERVH LTR7 have donated cell-specific transcription initiation signals to many of these lincRNAs.

Consistent with this notion, we noticed a strong correlation between HERVH elements and histone modifications that is restricted to pluripotent cells. Trimethylation of lysine 4 on histone 3 (H3K4me3) plays a major role in activating transcription in ESCs [67]. We found that HERVH elements in lincRNAs are the most significantly enriched of all TE families for H3K4me3 ChIP-Seq reads generated by ENCODE in both H1-hESCs (*P *~ 0) and H7-hESCs (*P *~ 0). Enrichment of reads could be found at nearly all HERVH elements in lincRNAs - peak calls overlapped 92% (Additional file 6). In contrast, HERVH elements in lincRNAs are depleted for H3K4me3 in GM12878 cells, where expression of HERVH-lincRNAs is far reduced. While H3K4me3 typically has a signature bimodal peak surrounding gene TSSs (Figure S5 in Additional file 1), coverage of HERVH-lincRNAs tends to be downstream of the TSS (Figure 4d), suggesting that primarily the HERVH-int downstream of the LTR is methylated.

Similar to H3K4me3, occupancy of the transcription factor SP1 also correlates with HERVH-lincRNA expression in stem cells. Prior work discovered that SP1 acts as a transcriptional activator for HERVH by binding to the 5' LTR [68]. We found that SP1 is ubiquitously expressed across many cell types with similar FPKMs in H1-hESC and GM12878 (Figure S15a in Additional file 1). Using SP1 ChIP-Seq generated by ENCODE, we verified that SP1 occupies the TSSs of proteins and lincRNAs in both cell types (Figure S15b, c in Additional file 1). In H1-hESCs, the LTRs of HERVH-lincRNAs are enriched for SP1 ChIP-Seq reads (*P *~ 0) and 93% overlap an SP1 peak call (*P *4e-67). In contrast, SP1 ChIP-Seq reads are depleted at HERVH-lincRNAs in GM12878 (*P *6e-69). Accordingly, SP1 coverage peaks at the TSS of HERVH-lincRNAs in H1-hESC, but not GM12878 (Figure 4e).

We also detected occupancy of the pluripotency transcription factors Oct4 and Nanog on HERVH in lincRNAs via enrichment of reads (Oct4 2.1-fold, *P *~ 0; Nanog 7.3-fold, *P *~ 0) and overlap with peak calls (Oct4 73%, *P *2e-17; Nanog 91%, *P *2e-16), suggesting that many of these lincRNAs have been fully incorporated into pluripotency regulatory networks (Figure S16 in Additional file 1).

Finally, HERVH-lincRNAs have a number of interesting evolutionary properties. First, HERVH elements associated with lincRNAs are evolutionarily younger than HERVH elements genome-wide. We classified every HERVH element in the human genome by the earliest primate ancestor where the homologous region in that genome (mapped via BlastZ whole-genome alignments [69]) has a RepeatMasker-annotated HERVH (Figure S17a in Additional file 1). We found that HERVH elements associated with lincRNAs inserted more recently than other HERVH elements genome-wide (*P *1.6e-3). Second, in lincRNAs, the flanking LTR7 appears to be evolving slower than HERVH-int. lincRNA LTR7 annotations are significantly more similar to RepeatMasker's LTR7 consensus than are LTR7 annotations outside of lincRNAs (85.8% nucleotide identity versus 81.8%, *P *3.1e-4), unlike HERVH-int annotations, which are slightly less similar to the consensus in lincRNAs (83.5% versus 84.2%). In every primate genome, LTR7 is present at a greater proportion than HERVH-int in the mapped lincRNA HERVH elements (Figure S17b in Additional file 1); that is, the interior has more often been deleted or mutated beyond recognition.

Altogether, these observations suggest that HERVH insertions may have originated or altered 127 lincRNAs to have stem cell-specific expression by imparting transcriptional regulatory signals. Accordingly, the signal-heavy LTR is more robust to mutation than the HERVH interior.

#### Mouse TE-lincRNAs exhibit similar properties to human

**Figure 5:**
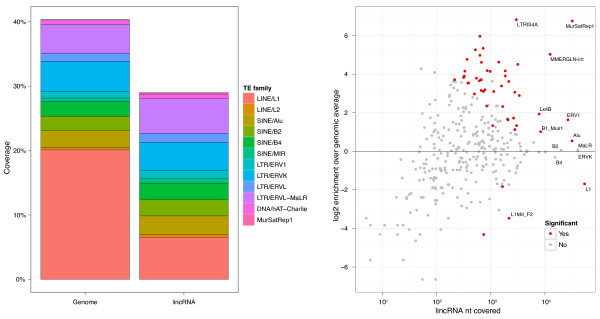
**Mouse lincRNAs share TE composition properties**. **(a) **Similar to the human genome, in a catalog of 981 mouse lincRNAs, TEs are depleted overall relative to the genomic background frequency, but still a substantial 33% of sequence is TE-derived. **(b) **TEs also exhibit biased composition in mouse lincRNAs, with strong L1 depletion and ERV1 enrichment, matching observations in human.

To assess whether the properties of TEs in lincRNAs observed in human carry over to other mammalian genomes, we performed the same analyses on a previously published catalog of mouse lincRNAs built from RNA-Seq of ESCs, lung fibroblasts, and neural precursor cells [1] and filtered down to 981 multi-exon > 200-nucleotide transcripts. The mouse genome contains fewer TEs than the human genome (41.4% versus 49.9%) and, accordingly, less mouse lincRNA sequence is TE-derived (33.0% versus 41.9%) (Figure 5a); 66% of these mouse lincRNAs contain some TE sequence, less than the 83% in human.

Mouse lincRNAs also comprise a nonrandom distribution of TEs. Similar to the human genome, L1 is depleted in lincRNAs (3.2-fold, *P *3e-25) and ERV1 is enriched (3.1-fold, *P *3e-16) - though the specific ERV1 families differ from human. In contrast to human, Alu elements are enriched 1.5-fold (*P *1.9e-6), and other SINEs, MIR, B2, and B4, differ insignificantly from the genome. An unknown repeat family named MurSatRep1 is enormously enriched 108-fold (*P *~ 0) and overlaps 81 lincRNAs. The ERV1 family MMERGLN-int, recently discovered to undergo significant reduction of DNA methylation between mouse sperm to zygote [70], is 33-fold enriched (*P *1.2e-130) and overlaps 10 lincRNAs.

TEs in mouse lincRNAs also display biased position and orientation. For example, ERVK associates with TSSs, visible as peaked coverage (Figure S18 in Additional file 1) and a significant preference for the sense orientation (78%, *P *1e-4). MurSatRep1 appears in the sense orientation in a striking 96% of its 81 lincRNAs (*P *4e-32).

Finally, we found several interesting relationships between the TE content of a lincRNA and its expression profile in these three cell types. Similar to human, TEs correlate with expression biases in mouse. TE-lincRNAs have significantly reduced expression in lung fibroblasts (*P *2e-23) and neural precursor cells (*P *3e-17) but increased expression in ESCs (*P *2e-10) relative to dTE-lincRNAs (Figure S19a in Additional file 1). The ERVK family has a particularly strong effect in ESCs; the FPKM geometric mean of 142 lincRNAs is 2.1-fold greater (Figure S19b in Additional file 1). In summary, although the specific TEs in mouse mostly differ from human, they associate with lincRNAs comparably to our observations in human, suggesting that the properties described here may be more broadly applicable to lincRNAs throughout the mammalian phylogeny.

## Discussion

It is now clear that there are many thousands of lincRNA transcripts encoded in the human genome that play critical functional roles across a spectrum of cellular processes [6-8,71-74]. However, lincRNA sequence properties and evolutionary origins are just emerging [1-3,5,21,22]. One intriguing hypothesis is that TEs have significantly shaped the noncoding transcriptome. By stochastically inserting around the genome, TEs may modify the regulation, sequence, and structure of existing lincRNAs and establish new lincRNA loci through their transcription promoting abilities. Here, we investigated this hypothesis by comprehensively characterizing the TE composition of lincRNAs and exploring correlations with their functional and evolutionary properties.

Indeed, we uncovered many new aspects of lincRNA biology related to TE content. lincRNAs contain a high proportion of TE-derived sequence, less than the genomic background but much greater than protein coding genes. The highly abundant LINE and SINE families are depleted in lincRNAs, indicating that they may be deleterious to lincRNA functions. Conversely, we observed a strong enrichment of many ERV families. ERVs also exhibit position and orientation biases, preferring the 5' end of lincRNA transcripts and sense orientation with the transcript, consequently placing their LTRs in proper position to promote transcription. This suggests that transposition of ERVs may play a role in lincRNA transcriptional regulation. Interestingly, although both are regulated by RNA polymerase II, this enrichment is unique to lincRNAs and absent at mRNA TSSs.

Exemplifying this phenomenon, we discovered that 127 HERVH-lincRNAs are strikingly enriched for HERVH LTR7 in the sense orientation at their TSSs and exhibit dramatic stem cell-specific expression, observed in both H1-hESCs and iPSCs. Consistent with the notion of TEs contributing promoter regulatory signals, the HERVH elements in these lincRNAs are highly enriched for transcription activation signals in ESCs, but not other cell types. Presence of the activating histone modification H3K4me3 and SP1, a transcription factor previously found to be critical for transcription of HERVH [68], suggests that lincRNAs can acquire the remnant regulatory signals of their comprising TEs. *Linc-ROR *is an example of a HERVH-lincRNA, which we previously showed modulates the reprogramming process from fibroblasts into iPSCs [52]. Similar to *linc-ROR*, we observed strong enrichment of the core pluripotency factors (Oct4 and Nanog) at the 127 HERVH-lincRNAs. Collectively, these data suggest that HERVH retrotransposition may have shaped pluripotency networks via lincRNA regulation.

We found that TEs partition lincRNAs into two classes with divergent properties; 1,531 lincRNAs are devoid of TEs (dTE-lincRNAs), unlike the majority of 7,710 lincRNAs that contain TEs (TE-lincRNAs). This classification of lincRNAs uncovered another example of TE content influencing lincRNA expression as TE-lincRNAs are less expressed in every tissue and cell line, particularly testis. The relative decrease in expression of TE-lincRNAs may be due to lasting effects of well-established TE silencing mechanisms in germline cells [75], which have been described in TE-derived promoters of protein coding genes [76]. Despite TE-lincRNAs exhibiting lower expression overall, Alu-lincRNAs are significantly more expressed in all tissues except testis. Whether this increase is attributable to a transcriptional or post-transcriptional regulatory effect of the Alu sequence remains to be determined.

dTE-lincRNAs also have greater evidence of conservation by substitution-based statistics than TE-lincRNAs (even after removal of TE content). The lower conservation levels of TE-lincRNAs is apparently permissive of function as there are many examples of important TE-lincRNAs, including *TUG1 *[54], *linc-ROR *[52], *PCAT-1 *[13], *SLC7A2-IT1A *[51], *BANCR *[77], and more. Further interpretation of the low conservation of TE-lincRNAs requires knowing the age of the lincRNAs and the evolutionary order of events of TE insertions and the origin of transcription of the loci. More specifically, (1) did a young lincRNA arise from previously neutrally evolving sequence containing TEs or (2) did the lincRNA exist first and evolve rapidly via TE insertions?

The first scenario is highly plausible. Furthermore, we found evidence that TE insertions may have even played a role in creating those new lincRNAs. Retrotransposons contain promoters to transcribe the element, but, as selfish genomic parasites, typically mutate freely after insertion. This arrangement provides an opportunity for a new lincRNA to arise in the region downstream of an intergenic retrotransposon insertion where the sequence would usually have been evolving neutrally. In our data, we found many examples of TEs associating with lincRNA TSSs; a number of families, particularly ERV LTRs, peak in coverage at the TSS with biased orientation matching the known promoter direction. Whether these TE insertions truly spawned novel lincRNAs or simply donated an alternative TSS to an existing lincRNA will be the focus of future comparative transcriptome analyses. Nonetheless, it appears that TEs may lend regulatory signals to these lincRNAs, exemplified by the stem cell-specific expression of HERVH-lincRNAs.

The second scenario - a TE insertion altering a previously existing lincRNA - may also often occur. One hypothesis regarding lincRNA function and evolution proposes a language of independent, small sequence-structure domains [72]. Thus, lincRNAs may be resilient to mutations and TE insertions that avoid altering the resident domains. An intriguing follow-up question is whether some TE-derived sequence in lincRNAs may itself be functional. Recent research has described a number of groundbreaking examples of TEs in DNA affecting transcriptional regulation - for example, by distributing transcription factor binding site motifs inherent in the element throughout the genome [35-38]. Given the prevalence and biased composition of TEs in lincRNAs, it is tempting to hypothesize that TEs transcribed into lincRNAs may function analogously in post-transcriptional processes. For example, perhaps some TEs inherently contain binding sites for RNA binding proteins or interact with nucleic acids via sequence complementarity. Indeed, some evidence already exists for this model - Alu elements in lncRNAs can bind matching Alu elements in the 3' UTRs of mRNAs to form a binding site for Staufen 1 to initiate RNA decay [50]. Furthermore, TEs have been shown to act in a variety of post-transcriptional processes regulating mRNAs, such as RNA editing [78,79], stability [80], and translation efficiency [46,81]. To comprehensively explore the possibility of additional functional TE sequence in lincRNAs, more experimental data are needed.

More definitive answers to the evolutionary and regulatory questions raised by this study will require additional computational and experimental analyses. Specifically, this will require deep coverage RNA-Seq datasets to annotate lincRNA loci across primates and eutherian mammals. Such data would generalize trends in the TE composition of lincRNAs and reveal how lineage-specific TEs such as ERVs have shaped transcriptional regulation at lincRNA loci. Future experimental work will focus on exploring the functional role of TE sequence in lincRNAs through detailed mapping of lncRNA molecular interactions. In the meantime, it is now clear that TEs have significantly shaped the noncoding RNA landscape.

## Materials and methods

### Materials

We built the lincRNA catalog used in this analysis using RNA-Seq experiments from 28 different tissues and cell lines (Additional file 4) and UCSC, RefSeq, and GENCODE v4 annotations on the human genome assembly Hg19. We annotated transposons using RepeatMasker [82] on Hg19 with the RepBase repeat library 20110920 after subtracting non-coding RNA, satellite, low complexity, and simple repeats [83]. Protein coding transcript analyses were performed on UCSC annotations.

### lincRNA catalog

We mapped RNA-Seq reads to the Hg19 reference genome using the spliced alignment software TopHat [84]. We assembled transcripts for each tissue or cell line individually using Cufflinks [57]. We estimated gene abundance using Cuffdiff, simultaneously normalizing all libraries with the geometric mean normalization option. Similarly to a recent lncRNA catalog release [2], we implemented a set of filters for the assembled transcripts (Additional files 7, 8 and 9). To overcome transcriptional noise, we required two or more exons, length greater than 200 bp, and abundance estimate greater than 1 FPKM in at least one of the tissues or cell lines. Next, we removed transcripts with evidence of protein coding potential via either overlap with a UCSC/RefSeq/GENCODE v4 protein annotation or a Phylo-CSF score > 100 [85]. The threshold of 100 was found to correspond to a 10% false negative rate for known lncRNAs and 15% false positive rate for protein coding genes [2]. We also removed transcripts overlapping UCSC-annotated tRNA, rRNA, and small RNAs. We added back lncRNA annotations from UCSC/RefSeq/GENCODE v4 because these typically have additional experimental validation. However, we removed all transcripts antisense to protein annotation or overlapping a GENCODE v7 pseudogene to separate out these different classes of lncRNA. Finally, we analyzed only the isoform for each gene locus that had the greatest FPKM geometric mean across all tissues and cell lines (Additional file 8). We used the software package BEDtools extensively in this pipeline and overall analyses [86].

### Multi-mapping reads

Given the focus in this analysis on repetitive regions, we paid careful attention to the difficult issue of RNA-Seq reads mapping to multiple genomic positions [87]. We limited the number of alignments per read to 20. Cufflinks assembles multi-mapping reads in every aligning position, but discards any transcript consisting of greater than 50% multi-mapping reads. Thus, combined with the requirement of multiple exons, the evidence required for a transcript to be included in our catalog is substantial. To quantify expression, Cufflinks performs an initial FPKM estimation procedure in order to more accurately distribute multi-mapping reads in a second iteration. Nevertheless, in statistical comparisons, we imposed a minimum FPKM of 0.5 in order to ignore expression differences at very low levels that may be artifacts from multi-mapping reads spreading a small amount of supposed expression to quiescent transcripts.

To further validate the transcript assemblies around repeats, we re-assembled the reads from H1-hESC using only uniquely mapping reads. Differences between the two assemblies were minimal. Most differences were additional transcripts in the uniquely mapped assembly that failed the multi-mapping read proportion threshold in the original assembly but actually do have substantial support. Thus, we conclude high confidence in our transcript assemblies even in the presence of repeats.

### Statistical tests

To test for enrichment and depletion of specific TEs in various annotation sets, such as the lincRNA catalog, we implemented a shuffling procedure. More specifically, we shuffled the annotations (while freezing the TEs) and recomputed the statistic of interest 100 times. We fit a normal distribution to these null samples and computed *P*-values from the parameterized normal cumulative density function.

For all comparisons of two sets of values where we were interested in whether one set was greater than another, we used a Mann-Whitney rank sum test [88]. This included comparisons between TE-lincRNA and dTE-lincRNA length, exons/transcript, isoforms/gene, abundance estimates, and conservation scores.

To test for enrichment of ChIP-Seq reads in TEs, we used a binomial test modeling each read as a sample from a Bernoulli distribution where the success probability is proportional to the size of the TE family over the size of the alignable genome.

*P*-values in all experiments were corrected for multiple hypothesis testing using Benjamini and Hochberg's false discovery rate procedure [89].

### ChIP-Seq analysis

We downloaded fastq files of ChIP-Seq reads generated by the ENCODE consortium from UCSC (Additional file 6) and analyzed the data two different ways in order to avoid multi-mapping read biases. First, we mapped the reads to the genome using Bowtie [90] with the --best option to return the single best alignment per read. Using these alignments, we computed enrichment of reads within TEs and plotted read coverage at TSSs (normalized by subtraction of control sequencing coverage). In TE-specific TSS coverage plots, only genes containing that TE in a promoter region 2,000 nucleotides upstream and 200 nucleotides downstream were considered. The choice of one alignment per read for these meta-feature analyses should mitigate multi-mapping read challenges. Second, we remapped the reads allowing up to 20 alignments and called peaks using the AREM software package, which implements an iterative algorithm to optimally allocate multi-mapping reads built on top of the MACS method [91,92].

## Abbreviations

ChIPm: chromatin immunoprecipitation; ERV: endogenous retrovirus; ESC: embryonic stem cell; FPKM: fragments per kilobase per million; H3K4me3: trimethylation of lysine 4 on histone 3; iPSC: induced pluripotent stem cell; lincRNA: long intergenic noncoding RNA; LINE: long interspersed nuclear element; lncRNA: long noncoding RNA; LTR: long terminal repeat; SINE: short interspersed nuclear element; TE: transposable element; TSS: transcription start site; UTR: untranslated region.

## Competing interests

The authors declare that they have no competing interests.

## Authors' contributions

DK and JR conceived the study and wrote the manuscript. DK carried out the analyses. All authors have read and approved the manuscript for publication.

## Supplementary Material

Additional file 1 **Supplementary results and figures**. [94] Click here for file

Additional file 2 **TE content of lincRNAs**. Excel table describing TE content of lincRNAs and statistical analysis. Click here for file

Additional file 3 **TE content of protein coding genes**. Excel table describing the TE content of protein coding genes and statistical analysis. Click here for file

Additional file 4 **RNA-Seq data**. Excel table describing RNA-Seq datasets used to build lincRNAs and estimate abundances. Click here for file

Additional file 5 **TEs in lincRNAs orientation statistics**. Excel table describing orientation statistics of TEs in lincRNAs. Click here for file

Additional file 6 **ChIP-Seq data**. Excel table describing ChIP-Seq datasets used to study HERHV-lincRNAs. Click here for file

Additional file 7 **GTF file describing our full lincRNA catalog**. Click here for file

Additional file 8 **GTF file describing only the most expressed isoforms of our lincRNA catalog**. Click here for file

Additional file 9 **Cufflinks output file describing abundance estimates for our full lincRNA catalog**. Click here for file
