# Supplementary material for: Transposable elements reveal a stem cell-specific class of long noncoding RNAs
Source: Genome Biol. 2012 Nov 26;13(11):R107. doi: 10.1186/gb-2012-13-11-r107 (PMC3580499; doi:10.1186/gb-2012-13-11-r107)
Supplement: Additional file 1 — Supplementary results and figures. [94] [file gb-2012-13-11-r107-S1.DOCX]

**Supplementary Results**

**TEs in promoters**

Due to their generally low abundance[1], assembly of complete lincRNA transcripts from RNA-Seq reads is difficult. Nevertheless, we aimed to infer the positions of the TSSs and promoters for assembled lincRNAs to investigate a role for TEs in lincRNA transcriptional regulation. Validating our TSS predictions, we observed similar profiles of the known activating histone modification H3K4me3 for lincRNAs and protein coding genes (Supplementary Figure 4). We compared and contrasted the TE composition of protein coding and lincRNA promoters.

The upstream promoter regions of protein coding genes are known to harbor TEs, which in some cases shape transcriptional regulation[2-4]. We confirmed that TEs comprise 32% of protein promoter sequence (defined as 2000 nt upstream of the TSS) and 40% of lincRNA promoter sequence (Figure 1a, Supplementary Figure 2). Overall, protein and lincRNA promoter regions are much more similar in TE composition than their respective transcripts.

However, there are notable differences. The SINE families Alu and MIR are enriched in protein promoters, as noted previously[3, 5], but are nearer to genomic background rates in lincRNA promoters. Alternatively, ERV1 is depleted in protein promoters, but enriched 1.5 fold (P 3e-21) in lincRNA promoters. Particular ERV1 families have been shown to contain transcription factor binding sites[6-8], and many of these families are enriched in lincRNA promoters, such as 2.0 fold enrichment (P 3.6e-3) of MER61 containing the p53 binding motif (Supplementary Table 2)[6]. The known ability of ERV1 LTRs to initiate transcription may play a role in their prevalence, as they are 3.1 fold enriched at lincRNA TSSs (P 4e-97). Collectively, although protein and lincRNA promoters have broadly similar TE content, they exhibit intriguing differences.

**Supplementary Figures**

**Supplementary Figure 1: LincRNAs are similar to a recently studied catalog**

Our catalog of 9,241 lincRNAs shares a number of important properties with a previously published catalog by Cabili et al[1]. (a) The distributions of transcript length are comparable, though the geometric mean transcript length in our catalog is 1053 nt, larger than 715 nt for the Cabili catalog. Splicing complexity is similar between the sets: (b) There are 2.8 exons per transcript (2.9 in Cabili) and (c) 2.3 isoforms per gene in our catalog (2.3 in Cabili). (d) Our lincRNA catalog maintains the substantial tissue-specificity (computed as in Cabili et al) relative to proteins observed previously. (e) The lincRNAs are slightly more conserved than the Cabili catalog based on PhyloP scores, plotted here as a cumulative distribution.

**Supplementary Figure 2: TE composition of human genes**

Similar to Figure 1a, TE composition of various features related to human lincRNAs and protein coding genes.

**Supplementary Figure 3: Basic statistics of TEs in lincRNAs**

(a) Histogram bars represent the number of lincRNAs covered at certain proportions by TEs. 1,531 (17%) of 9,241 lincRNAs are completely devoid of TEs, but most contain some TE sequence—33% is the median TE coverage of lincRNA transcripts. Plotted in red on the right is the median lincRNA length in each coverage bin. TE coverage correlates with transcript length, though the relationship is not monotonically increasing. (b) Histogram bars represent the number of lincRNAs containing a given number of unique TEs. The mean number of TEs per lincRNA is 2.8. Plotted in red on the right is the median lincRNA length in each bin. The number of unique TE families per lincRNA correlates very strongly with transcript length.

**Supplementary Figure 4: H3K4me3 ChIP-Seq TSS coverage**

Validating our lincRNA TSS annotations, coverage of H3K4me3 ChIP-Seq reads (normalized by control) in H1-hESC at the TSS of our (b) lincRNAs matches the bimodal peak pattern seen for (a) protein coding genes.

**Supplementary Figure 5: TE-lincRNA transcript structure**

(a) TE-lincRNAs are longer than dTE-lincRNAs, which is unsurprising given that longer lincRNAs have more opportunity to collect TE insertions. TE-lincRNAs also have greater splicing complexity than dTE-lincRNAs with (b) 2.88 (versus 2.59) exons/transcript and (c) 2.35 (versus 2.09) isoforms/gene.

**Supplementary Figure 6: TEs correlate with lincRNA expression**

(a) In our compendium of RNA-Seq from 29 different tissues and cell lines, TE-lincRNAs are less expressed than dTE-lincRNAs in all tissues and cell lines, exemplified most significantly here in testis as the cumulative distribution of log2(FPKM+0.25). (b) In contrast, lincRNAs containing Alu elements are more expressed in all tissues and cell lines except testis, exemplified here in H1-hESCs.


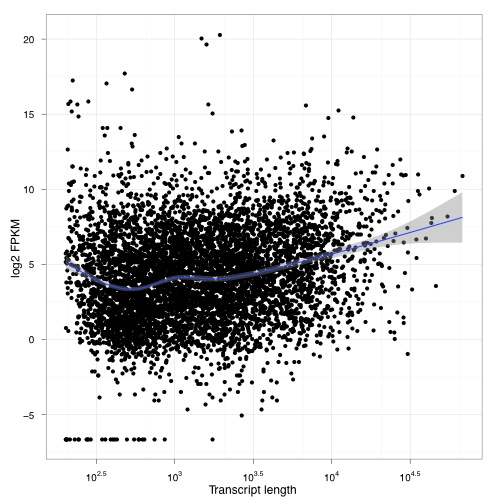


**Supplementary Figure 7: LincRNA transcript length correlates with expression**

LincRNA transcript length correlates positively (Spearman 0.174) with its maximum FPKM across the 29 tissues and cell lines analyzed. This correlation strengthens the conclusion that TE-lincRNAs (which tend to be much longer than dTE-lincRNAs) are less expressed.

**Supplementary Figure 8: TE-lincRNA conservation**

We analyzed conservation score distributions assigned by PhastCons and PhyloP based on the placental mammal phylogeny. For PhastCons, the score reflects a probability of the nucleotide having evolved in a conserved versus neutral state. For PhyloP, positive scores indicate negative selection (conservation) on the sequence, and negative scores indicate positive selection. (a) Conservation of TEs in lincRNAs is nearly indistinguishable from TEs genome-wide. (b) dTE-lincRNAs are more conserved than TE-lincRNAs. (c) Within TE-lincRNAs, non-TE sequence is slightly more conserved than TE sequence. Notably, TE sequence has much lower variance of PhyloP scores than non-TE sequence, which, accordingly, has a greater proportion of nucleotides under both negative and positive selection.

**Supplementary Figure 9: Alu coverage peaks at lincRNA 3’ ends**

We plotted TE coverage downstream of lincRNA and protein coding gene 3’ ends. In agreement with previous observations of polyadenylation signals in Alu elements, (a) Alu coverage peaks at the 3’ ends of lincRNAs. (b) This is especially true for the specific family AluY. (c,d) In contrast, coverage peaks are absent at the 3’ ends of protein coding genes.


**Supplementary Figure 10: LTR coverage peaks at lincRNA TSSs**

In the left column, we plotted LTR coverage throughout the promoter, leading up to the lincRNAs’ TSS. In the right column, we plotted LTR coverage in the interior of the lincRNAs, which are broken up into 100 uniformly sized bins to normalize for transcript length. (a) LTR7, (b) LTR12, and (c) THE1 all display a clear pattern of increasing coverage through the promoter leading to a peak at the TSS before declining into the lincRNA.

**Supplementary Figure 11: ERV1 LTR coverage does not peak at protein TSSs**

Similar to Figure 3, we plotted L1, Alu, and ERV1 LTR coverage at (a) lincRNA TSS where ERV1 LTRs peak in coverage and (b) protein coding gene TSS where they do not.

**Supplementary Figure 12: LTR coverage does not peak at protein TSSs**

Similar to Supplementary Figure 10, in the left column, we plotted LTR coverage throughout the promoter, leading up to the TSSs of protein coding genes. In the right column, we plotted LTR coverage in the interior of the protein coding genes, which are broken up into 100 uniformly sized bins to normalize for transcript length. In contrast to lincRNAs, (a) LTR7, (b) LTR12, and (c) THE1 do not display a coverage peak at TSSs of protein coding genes.

**Supplementary Figure 13: HERVH-mRNA expression is not stem cell specific**

Similar to Figure 4b,c, we analyzed the expression of 35 protein coding genes that contain HERVH in their exonic sequence or a 2 kb upstream promoter. In contrast to HERVH-lincRNAs, they do not display specific and high expression in pluripotent cells. (a) Expression of these protein coding genes (measured as log2(FPKM+0.25)) across cell types (rows) is not specific to any particular cell type. (b) Furthermore, their H1-hESC expression is indistinguishable from protein coding genes devoid of HERVH.

**Supplementary Figure 14: HERVH-mRNA regulatory signals are not ESC-specific**

Similar to Figure 4d,e, we plotted ChIP-Seq read coverage (normalized by control) of (a) H3K4me3 at HERVH-lincRNA TSSs, (b) SP1 at HERVH-lincRNA TSSs, (c) H3K4me3 at HERVH-mRNA TSSs, and (d) H3K4me3 at HERVH-mRNA TSSs. Unlike HERVH-lincRNAs where the presence of the regulatory signals is specific to H1-hESCs, the regulatory signals are detectable in both cell types at mRNA TSSs.

**Supplementary Figure 15: SP1 properties**

(a) The transcription factor SP1 is ubiquitously expressed in our RNA-Seq compendium and was quantified at near equal levels in H1-hESC and GM12878. We produced the plot using CummeRbund[9]. ChIP-Seq read coverage (normalized by control) around (b) proteins and (c) lincRNAs shows an association with TSSs in both cell types.

**Supplementary Figure 16: Oct4 and Nanog HERVH-lincRNA binding**

ChIP-Seq read coverage (normalized by control) of the pluripotency-regulating transcription factors (a) Oct4 and (b) Nanog peaks at the TSSs of HERVH-lincRNAs.

**Supplementary Figure 17: HERVH mapped in primates**

(a) Mapping HERVH throughout the primate phylogeny shows that HERVH elements in lincRNAs are younger than HERVH elements elsewhere. On the left, the bars represent the percentage of human HERVH elements present in that genome. E.g. 45% of human lincRNA HERVH elements are present in orangutan. On the right, the bars represent the percentage of human HERVH elements that first arose in that genome. E.g. 9% of human lincRNA HERVH elements most distantly appear in orangutan. (b) HERVH-int mutates faster than LTR7. The y-axis represents the percentage of total nucleotides from the human HERVH occurrences that appear in each genome, assuming the original insertion included the full consensus HERVH.

**Supplementary Figure 18: ERVK associates with mouse lincRNA TSSs**

ERVK demonstrates a positional preference for lincRNA TSSs. (a) We plotted TE coverage throughout the promoter, leading up to the TSS of the lincRNA. (b) We plotted TE coverage in the interior of the lincRNAs, which were divided into 100 bins to normalize for transcript length.

**Supplementary Figure 19: Mouse TE-lincRNA expression patterns**

We plotted the cumulative distributions of log2(FPKM+0.25) of various classes of mouse TE-lincRNAs. (a) TE-lincRNAs are significantly more expressed than dTE-lincRNAs in ESCs and significantly less expressed in LFs and NPCs. (b) This phenotype is especially present in lincRNAs containing the ERVK family.

**Supplementary References**

1. Cabili MN, Trapnell C, Goff L, Koziol M, Tazon-Vega B, Regev A, Rinn JL: **Integrative annotation of human large intergenic noncoding RNAs reveals global properties and specific subclasses**. *Genes Dev* 2011, **25**:1915–1927.

2. Jordan IK, Rogozin IB, Glazko GV, Koonin EV: **Origin of a substantial fraction of human regulatory sequences from transposable elements.** *Trends in Genetics* 2003, **19**:68–72.

3. Thornburg BG, Gotea V, Makałowski W: **Transposable elements as a significant source of transcription regulating signals.** *Gene* 2006, **365**:104–110.

4. Feschotte C: **Transposable elements and the evolution of regulatory networks**. *Nat Rev Genet* 2008, **9**:397–405.

5. Polak P, Domany E: **Alu elements contain many binding sites for transcription factors and may play a role in regulation of developmental processes.** *BMC Genomics* 2006, **7**:133.

6. Wang T, Zeng J, Lowe CB, Sellers RG, Salama SR, Yang M, Burgess SM, Brachmann RK, Haussler D: **Species-specific endogenous retroviruses shape the transcriptional network of the human tumor suppressor protein p53.** *Proc Natl Acad Sci* 2007, **104**:18613–18618.

7. Bourque G, Leong B, Vega VB, Chen X, Lee YL, Srinivasan KG, Chew J-L, Ruan Y, Wei C-L, Ng HH, Liu ET: **Evolution of the mammalian transcription factor binding repertoire via transposable elements**. *Genome Res* 2008, **18**:1752–1762.

8. Kunarso G, Chia N-Y, Jeyakani J, Hwang C, Lu X, Chan Y-S, Ng H-H, Bourque G: **Transposable elements have rewired the core regulatory network of human embryonic stem cells.** *Nat Genet* 2010, **42**:631–634.

9. **CummeRbund** [http://compbio.mit.edu/cummeRbund/].
